# Molecular Characterization of Retinoic Acid Receptor CgRAR in Pacific Oyster (*Crassostrea gigas*)

**DOI:** 10.3389/fphys.2021.666842

**Published:** 2021-04-08

**Authors:** Kaidi Jin, Qianqian Jin, Zhongqiang Cai, Baoyu Huang, Lei Wei, Meiwei Zhang, Wen Guo, Yaqiong Liu, Xiaotong Wang

**Affiliations:** ^1^School of Agriculture, Ludong University, Yantai, China; ^2^Changdao Enhancement and Experiment Station, Chinese Academy of Fishery Sciences, Changdao, China; ^3^Center for Mollusc Study and Development, Marine Biology Institute of Shandong Province, Qingdao, China

**Keywords:** *Crassostrea gigas*, retinoic acid receptor, retinoid X receptor, molecular characterization, retinoic acid

## Abstract

Retinoic acid (RA) signaling pathways mediated by RA receptors (RARs) are essential for many physiological processes such as organ development, regeneration, and differentiation in animals. Recent studies reveal that RARs identified in several mollusks, including Pacific oyster *Crassostrea gigas*, have a different function mechanism compared with that in chordates. In this report, we identified the molecular characteristics of CgRAR to further explore the mechanism of RAR in mollusks. RT-qPCR analysis shows that CgRAR has a higher expression level in the hemocytes and gonads, indicating that CgRAR may play roles in the processes of development and metabolism. The mRNA expression level of both CgRAR and CgRXR was analyzed by RT-qPCR after injection with RA. The elevated expression of *CgRAR* and *CgRXR* was detected upon all-*trans*-RA (ATRA) exposure. Finally, according to the results of Yeast Two-Hybrid assay and co-immunoprecipitation analysis, CgRAR and CgRXR can interact with each other through the C-terminal region. Taken together, our results suggest that CgRAR shows a higher expression level in gonads and hemocytes. ATRA exposure up-regulates the expression of CgRAR and CgRXR. Besides, CgRAR can interact with CgRXR to form a heterodimer complex.

## Introduction

Retinoic acid (RA), an important hormone derived from vitamin A, plays crucial roles in regulating many development and differentiation processes, such as axial patterning, tissue formation, nervous system development, and regeneration (Das et al., 2014; Ghyselinck and Duester, 2019; Pawlikowski et al., 2019). It has been reported that RA-activated responses are mediated by the RA receptors (RARs) and the retinoid X receptors (RXRs) in vertebrates (Balmer and Blomhoff, 2002; Gutierrez-Mazariegos et al., 2014; Ghyselinck and Duester, 2019). Both RARs and RXRs belong to the steroid hormone/thyroid hormone nuclear receptor superfamily, containing a well-defined DNA-binding domain (DBD) and a C-terminal ligand-binding domain (LBD) (Bourguet et al., 2000; Le Maire and Bourguet, 2014).

Many studies have revealed that the RAR gene undergoes duplications through evolution. As a result, there are three paralogous RAR genes that exist in most vertebrates, including RARα, RARβ, and RARγ (Bastien and Rochette-Egly, 2004; Escriva et al., 2006). In vertebrates, RARs interact with RXRs to form different combinations of a heterodimer. There are several RA isomers, including all-*trans*-RA (ATRA), 9-*cis*-RA (9cRA), and 13-*cis*-RA. ATRA is the primary ligand sensed by RAR–RXR heterodimers during the development process (Mic et al., 2003; Cunningham and Duester, 2015). After being combined with ATRA, RAR–RXR heterodimers regulate transcription by combining with RA response elements (RAREs) in the regulatory regions of target genes (Kastner et al., 1995; Bastien and Rochette-Egly, 2004). In addition, RARs identified in urochordates *Polyandrocarpa misakiensis* and cephalochordate *Branchiostoma lanceolatum* can form a heterodimer with RXRs and combine with RA to activate transcription of target genes, with a similar function to that in vertebrates (Kamimura et al., 2000; Escriva et al., 2006).

It was once thought that RA signaling was unique to chordate animals for a long time until the signaling pathway was found in non-chordate animals, including ambulacrarians and lophotrochozoans, over the past one or two decades (Bertrand et al., 2004; Gutierrez-Mazariegos et al., 2014). Many studies focused on RA signaling pathway and the function of RARs in mollusks over the years. In *Lymnaea stagnalis*, RA modifies invertebrate electrical synapses of central neurons and function in the formation and modulation of invertebrate central synapses (Rothwell et al., 2017). Disruption of RA signaling in *Lymnaea* embryos using RAR antagonists resulted in abnormal eye and shell development (Carter et al., 2015). In addition, RA reduces intracellular calcium levels rapidly and affects calcium signaling in adult molluscan neurons of *L. stagnalis* (Vesprini et al., 2015). In *Thais clavigera* and *Nucella lapillus*, RXR is involved in the organotin-induced development of imposex (Nishikawa et al., 2004; Castro et al., 2007). Moreover, RXR identified from *Biomphalaria glabrata* can combine with 9cRA and activate the transcription of targets genes (Bouton et al., 2005). TcRAR and NlRAR, identified from *T. clavigera* and *N. lapillus*, respectively, can interact with RXR to form heterodimers but appear not to be activated by RA when detecting the transcription activity in mammalian cells (Urushitani et al., 2013; Juliana et al., 2014). In *Crassostrea gigas*, both *in silico* analysis and molecular experiments indicated that CgRXR shows high potential to combine with RA, while CgRAR loses the ability to interact with natural or synthetic RA ligands. In addition, RA can activate the transcriptional activity of CgRXR but not CgRAR (Vogeler et al., 2017; André et al., 2019; Huang et al., 2020). Thus, effects of RA on mollusks might be RAR independent. RAR may have different functions in these mollusks, including *C. gigas.*

Pacific oyster *C. gigas* is an important marine shellfish in the world, with great ecological and economic significance. It is of great significance to explore the RA signaling mechanism and RAR function in *C. gigas*. In the current report, we investigate the molecular characteristics of CgRAR. Tissue expression pattern and mRNA expression of CgRAR upon ATRA exposure were analyzed in *C. gigas* first. Then the subcellular localization of CgRAR was detected in HEK293T cells. To get a better understanding of the possible functions of CgRAR, the interaction between CgRAR and CgRXR was detected, and the results show that CgRAR interacts with CgRXR through the C-terminal region. Besides, both CgRAR and CgRXR can interact with themselves to form homodimers in yeast and mammalian cells.

## Materials and Methods

### Oyster Collection, Tissue Sampling, and Retinoic Acid Exposure

Oysters used in this study were collected from a local culture zone (Yantai, China), with an average shell length of 65 cm. All the oysters were acclimated in aerated seawater at 15–22°C for at least 1 week before experiment. For tissue expression analysis, mantle (Man), gill (Gil), adductor muscle (Amu), hemolymph (Hae), digestive gland (Dgl), gonad (Gon), and labial palps (Lpa) were collected from nine wild oyster individuals. For RA exposure analysis, 48 oysters were divided into two groups randomly. ATRA was dissolved in dimethylsulfoxide (DMSO); oysters in two groups were injected with DMSO and 2 μg/μl of ATRA and given a supplementary injection every 2 days for a total of five injections.

### RNA Extraction and Real-Time Fluorescence Quantitative PCR Analysis

Total RNA was isolated from different oyster tissues or RA-treated oysters using an RNA extraction kit (Tiangen). Briefly, tissue blocks were ground into a homogenate in liquid nitrogen, the cracking buffer was added to the homogenate, the supernatant was obtained by centrifugation, and subsequent operations were performed according to the kit instructions. cDNA synthesis using PrimeScript^TM^ RT Master Mix (TaKaRa) was performed according to the instructions. RT-qPCR analysis was performed using SYBR Premix Ex Taq II (TaKaRa) and a Bio-Rad CFX Connect PCR instrument. *RS18* was used as reference gene for normalization of gene expression. 2^–ΔΔCt^ method was used to calculate the relative expression level. Primers utilized for RT-qPCR are listed in Supplementary Table 1.

### Subcellular Localization Analysis

Full-length coding sequence (CDS) of CgRAR was cloned into *pEGFP-N1* plasmid (Clontech) and then transferred into HEK293T cells with Lipofectamine 3000 (Invitrogen) when the confluence of cells reached 60%. Transfected cells were fixed with 4% paraformaldehyde and then incubated with DAPI for 5 min to stain the nuclei 24 h after transfection. Laser-Scanning Confocal Microscopy System FluoView FV1000 (Olympus, Japan) was used to observe fluorescent signal. Primers used for fusion vector construction are listed in Supplementary Table 1.

### Yeast Two-Hybrid Assay

The yeast strain Yeast Two-Hybrid (Y2H) Gold (Clontech) was used to assess protein–protein interactions in this study. The CDS fragments of CgRAR, CgRXR, C-terminal of *CgRAR*, and C-terminal of *CgRXR* were cloned into *pGAD T7* and *pGBK T7* to generate *CgRAR-AD/BD*, *CgRXR-AD/BD*, *CgRAR^*C*^-AD/BD*, and *CgRXR^*C*^-AD/BD* fusion plasmids; fusion plasmids were co-transferred into Y2H Gold yeast strain according to the manufacturer’s instructions (Clontech). Primers used for fusion plasmids construction are listed in Supplementary Table 1. Yeast transformants were grown on SD/-Leu/-Trp double drop out (DDO) medium for 3–5 days and then screened on selective SD/-Leu/-Trp/-His/-Ade quadruple drop-out (QDO) medium with aureobasidin A (AbA) and X-α-gal.

### Co-immunoprecipitation Assay

The full-length CDSs of CgRAR and CgRXR were constructed into *pCMV-Myc* and *pCMS-flag* to generate the fusion vectors. Primers used for fusion vectors construction are listed in Supplementary Table 1. The fusion vectors were introduced into the HEK293T cells by Lipofectamine 3000 (Invitrogen). Thirty-six hours after transfection, proteins were extracted from co-transferred HEK293T cells with lysis buffer (Beyotime, China). Anti-flag magnetic beads (Sigma, United States) were used for co-immunoprecipitation (co-IP) as indicated. After the IP, beads were washed at least three times in wash buffer. Input samples were separated from the cell lysate without anti-flag magnetic beads. Samples were boiled for 5 min in 2× protein sodium dodecyl sulfate–polyacrylamide gel electrophoresis (SDS-PAGE) loading buffer (TAKAEA) and subjected to western blot (WB) analysis using anti-flag and anti-myc (Sigma, United States).

## Results

### Tissue Expression Pattern of CgRAR

**FIGURE 1:**
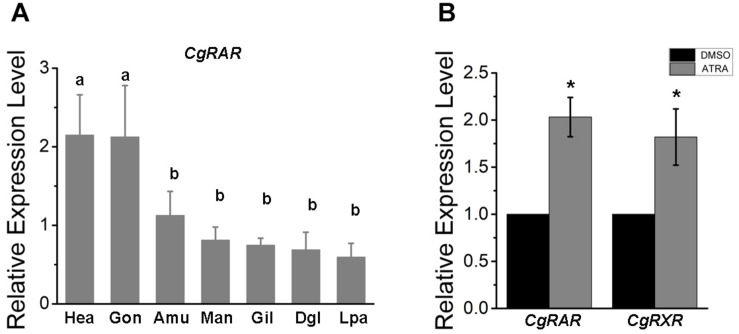
Relative expression of CgRAR in different tissues **(A)** and in response to retinoic acid (RA) treatment **(B)**. Relative mRNA expression levels of *CgRAR* and *CgRXR* was normalized to that of *CgRS18*. Bars represent means of three replicates ± SD (standard deviation). One-way ANOVA and Student’s *t*-test (*P < 0.05) were used for significance analysis in **(A,B)** respectively. Hae, hemolymph; Gon, gonad; Amu, adductor muscles; Man, mantle; Gil, gill; Dgl, digestive gland; Lpa, labial palps.

Transcriptional expression pattern of *CgRAR* was detected in different tissues isolated from healthy adult oysters under normal growing conditions, including the mantle, adductor muscle, gill, digestive gland, gonad, labial palp, and hemocytes. As a result, the *CgRAR* gene is widely expressed in all the detected tissues, with a higher expression in hemocytes and gonads (Figure 1A), suggesting that CgRAR may function in these tissues.

### The Expression of CgRAR Can Be Activated by ATRA

To explore the relationship between CgRAR and RA-induced responses, we performed RT-qPCR analysis to detect the relative expression level of CgRAR and CgRXR after ATRA treatment. As shown in Figure 1B, the transcript level of both CgRAR and CgRXR was up-regulated after ATRA injection. This result indicates that mRNA expression of CgRAR and CgRXR can be up-regulated by ATRA exposure.

### CgRAR Protein Mainly Localized in the Nucleus in HEK293T Cells

**FIGURE 2:**
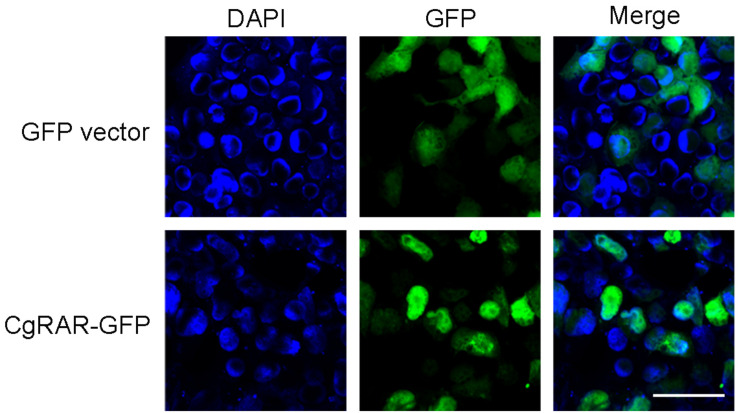
Subcellular localization of CgRAR in HEK293T cells. Confocal microscopy images of green fluorescent protein (GFP) (top) and CgRAR–GFP (bottom). HEK293T cells were transfected with *pEGFP-N1* and *CgRAR–GFP* respectively. Bar, 50 μm.

To reveal the subcellular localization of CgRAR protein, a green fluorescent protein (GFP)-tagged CgRAR was transferred into HEK293T cells. GFP plasmid was also transferred into HEK293T as control. As show in Figure 2, fluorescent single was observed on cytoplasm in GFP-transfected cells; in the cells transferred with CgRAR–GFP, fluorescent single was detected in the nucleus. This result suggests that CgRAR protein localized in the nucleus in HEK293T cells.

### CgRAR Physically Interacts With CgRXR

**FIGURE 3:**
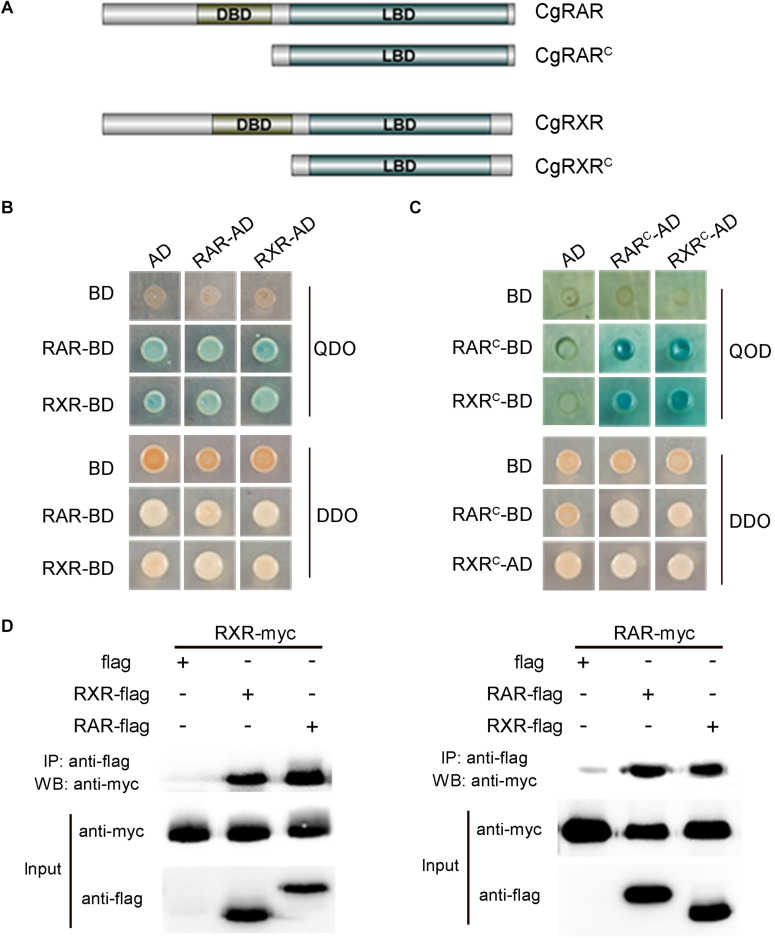
CgRAR physically interacts with CgRXR. **(A)** Representation of truncated CgRAR and CgRXR proteins. **(B)** Yeast two-hybrid assay for interactions between CgRAR and CgRXR protein. **(C)** Yeast two-hybrid assay for interactions between truncated CgRAR and CgRXR protein. AD and BD empty vectors were co-transformed as negative control. QDO/X/A, quadruple drop-out medium to test interaction; DDO, double drop-out as control. QDO, SD/-Trp/-Leu/-His/-Ade; DDO, SD-Leu-Trp; X, X-α-gal; A, aureobasidin A (AbA). **(D)** Co-immunoprecipitation assays verified the interaction between CgRAR and CgRXR protein. WB analysis using anti-myc and anti-flag on total protein extracts (“Input”) and on eluted proteins after immunoprecipitation (IP) with anti-flag magnetic beads.

To verify the interaction between CgRAR and CgRXR, a Y2H assay was carried out using the full-length CDSs of CgRAR and CgRXR first. Both CgRAR and CgRXR fusion vectors exhibit strong self-activation (Figure 3B). Thus, the DBD domain of CgRAR and CgRXR was deleted to generate CgRAR^*C*^-AD/BD and CgRXR^*C*^-AD/BD fusion vectors, respectively (Figure 3A). Yeast strain co-expressing CgRAR^*C*^ and CgRXR^*C*^ can be grown on QDO selection medium without self-activation (Figure 3C), indicating that CgRAR interacts with CgRXR through the C-terminal region. Co-IP analysis was also taken to confirm the interaction between CgRAR and CgRXR (Figure 3D). As a result, CgRAR was co-precipitated with CgRXR in HEK293T cells. In addition, both CgRAR and CgRXR can interact with themselves to form homodimers (Figures 3C,D). Taken together, these results demonstrate that CgRAR can either form a heterodimer by binding CgRXR or form a homodimer with itself in yeast and in HEK293T cells.

## Discussion

Retinoic acid is an important hormone playing critical roles in the processes of organogenesis, neuronal differentiation, and embryonic development in vertebrates (Duester, 2008; Kam et al., 2012; Janesick et al., 2015). The important roles of RA signaling were also found in physiological process in mollusks, such as maturation of neurons, imposex, and formation of central synapses (Nishikawa et al., 2004; Vesprini et al., 2015; Rothwell et al., 2017). RARs are the primary receptors that sense RA ligand and regulate target gene transcription in chordates. However, the RA signaling mechanism in non-chordate animals, especially in some mollusks, seems different from that in chordates (André et al., 2019).

To help predict the possible function of CgRAR, tissue expression pattern was first examined in adult oysters under normal indoor culture environment in this study. A universal distribution of CgRAR gene was detected in different tissues of oyster, with a higher expression in gonads and hemocytes (Figure 1A). Since both gonads and hemocytes are tissues closely related to the processes of reproduction, differentiation, and development, we speculated that the elevated expression of CgRAR in these two tissues suggested that CgRAR might be involved in the development process of *C. gigas*. In chordates, RARs and RXRs form a heterodimer to regulate the expression of target genes in the perception of ATRA (Niederreither and Dolle, 2008; Gutierrez-Mazariegos et al., 2014). A recent study revealed that CgRXR is widely expressed in all tissues examined in Pacific oyster, with the highest relative expression in the mantle and lowest in gonads (Huang et al., 2020). Thus, the main function of CgRAR and CgRXR may not be exactly the same.

All-*trans*-RA exposure activates the mRNA expression of both *CgRAR* and *CgRAR* in Pacific oyster (Figure 1B). This result indicates that transcriptions of both *CgRAR* and *CgRXR* can be respond to ATRA exposure. It is worth noting that activation at the transcriptional level does not mean that CgRAR and CgRXR proteins can sense and bind to ATRA ligands. Whether CgRAR and CgRXR function in this process and what role they play need to be further studied.

Both RARs and RXRs belong to the nuclear receptor family and can regulate target gene transcription in the nucleus in vertebrates (Bastien and Rochette-Egly, 2004). A recent study demonstrated that CgRXR localized in the nucleus in the human cell line (Huang et al., 2020). *CgRAR–GFP* fusion plasmid was constructed and transferred into HEK293T cells to explore the subcellular location of CgRAR. The results showed that CgRAR was also localized in the nucleus in the human cell line (Figure 2). Therefore, we speculate that CgRAR’s function in the nucleus may be to regulate the transcription of target genes as a transcription factor.

In vertebrates, RA–RAR–RXR complex targets RARE of downstream genes, activating or repressing gene transcription (Kastner et al., 1995; Cunningham and Duester, 2015). Several studies have shown that RAR gene identified in some mollusks, including *T. clavigera*, *Patella vulgata*, *N. lapillus*, and *C. gigas*, lose the ability to bind ligand RA. In addition, the transcriptional activity of these RARs cannot be activated by RA (Urushitani et al., 2013; Juliana et al., 2014; Vogeler et al., 2017; André et al., 2019). It is reported that the RAR–RXR heterodimer still has the ability to bind RAREs and recruit co-repressors to negatively regulate transcription in the absence of ligand (Niederreither and Dolle, 2008). In this study, the physical interaction between CgRAR and CgRXR was confirmed by Y2H assay and Co-IP analysis (Figure 3). Whether CgRAR or CgRAR–CgRXR heterodimer regulate target gene transcription in the nucleus remains to be further studied. Taken together, this study indicates that CgRAR localized in the nuclear and can interact with CgRXR to form a heterodimer complex. The transcription of CgRAR can respond to ATRA, and CgRAR may function during the development process. This work may help to better understand the possible functions of CgRAR and to provide data reference for the further research.

## Data Availability Statement

The original contributions presented in the study are included in the article/Supplementary Material, further inquiries can be directed to the corresponding author/s.

## Ethics Statement

Ethical review and approval was not required for the animal study because *Crassostrea gigas* is not an endangered or protected species and is not a vertebrate.

## Author Contributions

XW and YL designed the experiment. KJ, QJ, BH, LW, MZ, and YL carried out the experiments. ZC and WG contributed reagents, materials, and analysis tools. YL wrote the manuscript. XW supervised the study and revised the manuscript. All authors reviewed the manuscript. All authors contributed to the article and approved the submitted version.

## Conflict of Interest

The authors declare that the research was conducted in the absence of any commercial or financial relationships that could be construed as a potential conflict of interest.

## Supplementary Material

Supplementary Table 1 Primers used for RT-qPCR analysis and fusion vectors construction.
