# Supplementary material for: Molecular Characterization of Retinoic Acid Receptor CgRAR in Pacific Oyster (Crassostrea gigas)
Source: Front Physiol. 2021 Apr 8;12:666842. doi: 10.3389/fphys.2021.666842 (PMC8060629; doi:10.3389/fphys.2021.666842)
Supplement: Supplementary Table 1 — Primers used for RT-qPCR analysis and fusion vectors construction. [file Table_1.DOCX]

**Table S1.** Primers used for RT-qPCR analysis and fusion vectors construction.

| **Primer** | **Sequence (5′→3′)** | **Purpose** |
| --- | --- | --- |
| CgRAR-qF  CgRAR-qR  CgRXR-qF  CgRXR-qR  RS18-F  RS18-R  CgRAR-F  CgRAR-R CgRXR-F  CgRXR-R CgRAR^C^-F  CgRAR^C^-R CgRXR^C^-F  CgRXR^C^-R | GACAAGTCCTCGGGCTACCA  TGGACATTCCAGTGGCGTAG GAGAGAAGCTGTACAAGAGG  GAGCATCTATGTAGGTGTCTG  GCCATCAAGGGTATCGGTAGAC  CTGCCTGTTAAGGAACCAGTCAG  ATGAAGACGGACAGAATGAACC TCAAGGAATACACACATTCTCCTGCC  ATGGACCCATCAGAAATGG  TCAAGTGCTGCTTGGGGATG  AAGCCAAAGCTAGAGAACCC  TCAAGGAATACACACATTCTCCTGCC  CAGCGAGTGAAGGAGAAGG  TCAAGTGCTGCTTGGGGATG | RT-qPCR  RT-qPCR  RT-qPCR  RT-qPCR  RT-qPCR  RT-qPCR  generate RAR related vectors  generate RAR related vectors  generate RXR related vectors  generate RXR related vectors  generate CgRAR^C^-AD/BD  generate CgRAR^C^-AD/BD  generate CgRXR^C^-AD/BD  generate CgRXR^C^-AD/BD |
